# The preclinical assessment of XL388, a mTOR kinase inhibitor, as a promising anti-renal cell carcinoma agent

**DOI:** 10.18632/oncotarget.15620

**Published:** 2017-02-22

**Authors:** Zuquan Xiong, Yiwen Zang, Shan Zhong, Lujia Zou, Yishuo Wu, Shenghua Liu, Zujun Fang, Zhoujun Shen, Qiang Ding, Shanwen Chen

**Affiliations:** ^1^ Department of Urology, Huashan Hospital Affiliated to Fudan University, Shanghai, China; ^2^ Department of General Surgery, Huashan Hospital Affiliated to Fudan University, Shanghai, China

**Keywords:** renal cell carcinoma (RCC), mammalian target of rapamycin (mTOR), apoptosis, XL388, MEK-ERK

## Abstract

XL388 is a mammalian target of rapamycin (mTOR) kinase inhibitor. We demonstrated that XL388 inhibited survival and proliferation of renal cell carcinoma (RCC) cell lines (786-0 and A549) and primary human RCC cells. XL388 activated caspase-dependent apoptosis in the RCC cells. XL388 blocked mTOR complex 1 (mTORC1) and mTORC2 activation, and depleted hypoxia-inducible factor 1α (HIF1α) and HIF-2α expression in RCC cells. Yet, XL388 was ineffective in RCC cells with mTOR shRNA knockdown or kinase-dead mutation. Notably, XL388 was more efficient than mTORC1 inhibitors (rapamycin, everolimus and temsirolimus) in killing RCC cells. Further studies showed that activation of MEK-ERK might be a key resistance factor of XL388. Pharmacological or shRNA-mediated inhibition of MEK-ERK pathway sensitized XL388-induced cytotoxicity in RCC cells. *In vivo*, oral administration of XL388 inhibited in nude mice 786-0 RCC tumor growth, and its anti-tumor activity was sensitized with co-administration of the MEK-ERK inhibitor MEK162. Together, these results suggest that concurrent inhibition of mTORC1/2 by XL388 may represent a fine strategy to inhibit RCC cells.

## INTRODUCTION

Growing evidences have proposed a critical function of mammalian target of rapamycin (mTOR) in renal cell carcinoma (RCC) carcinogenesis and progression [1–4]. It is now known that mTOR lies in two different multi-protein complexes, including mTOR complex 1 (mTORC1) and mTORC2 [5–7]. The traditional mTOR1 is rapamycin sensitive, and is assembled with mTOR, Raptor, PRAS40 and several others [5–7]. The later-discovered mTORC2 is rapamycin insensitive, and is composed of mTOR, Rictor and Sin1 [5–7]. Both mTOR1 and mTORC2 are vital for promoting cancerous behaviors, such as cell proliferation, survival and migration as well as angiogenesis and apoptosis resistance [5–7].

Molecular-targeted therapy has drawn broad attentions in the RCC field [8]. For example, mTORC1 inhibitors (or rapalogs), including everolimus, temsirolimus, have been tested, which demonstrated clinical benefits in metastatic RCC patients [2, 9, 10]. The five-year survival of these patients has been improved after application of mTORC1 inhibitors [2, 9, 10]. Yet, there are several drawbacks when using these rapalogs in practice, including the incomplete inhibition of mTORC1, and more importantly, feed-back activation of oncogenic signaling pathways (*i.e*. AKT and ERK-MAPK) [11–14]. Therefore, mTOR kinase inhibitors, or the second generation of mTOR inhibitors, were developed [15]. These inhibitors, such as OSI-027, AZD-2014 and AZD-8055, block both mTORC1 and mTORC2 [11, 16]. In the preclinical cancer studies, these inhibitors have displayed promising anti-cancer efficiency [4, 17–22].

Very recent research efforts have characterized a novel, selective and orally-available ATP-competitive mTOR kinase inhibitor, named XL388 [23]. XL388 was shown to simultaneously block mTORC1 and mTORC2 activation [23, 24]. Its potential activity in RCC cells has not been tested thus far. In the current study, we show that XL388 exerts potent anti-RCC activity *in vitro* and *in vivo*.

## RESULTS

### XL388 inhibits RCC cell survival and proliferation

**Figure 1:**
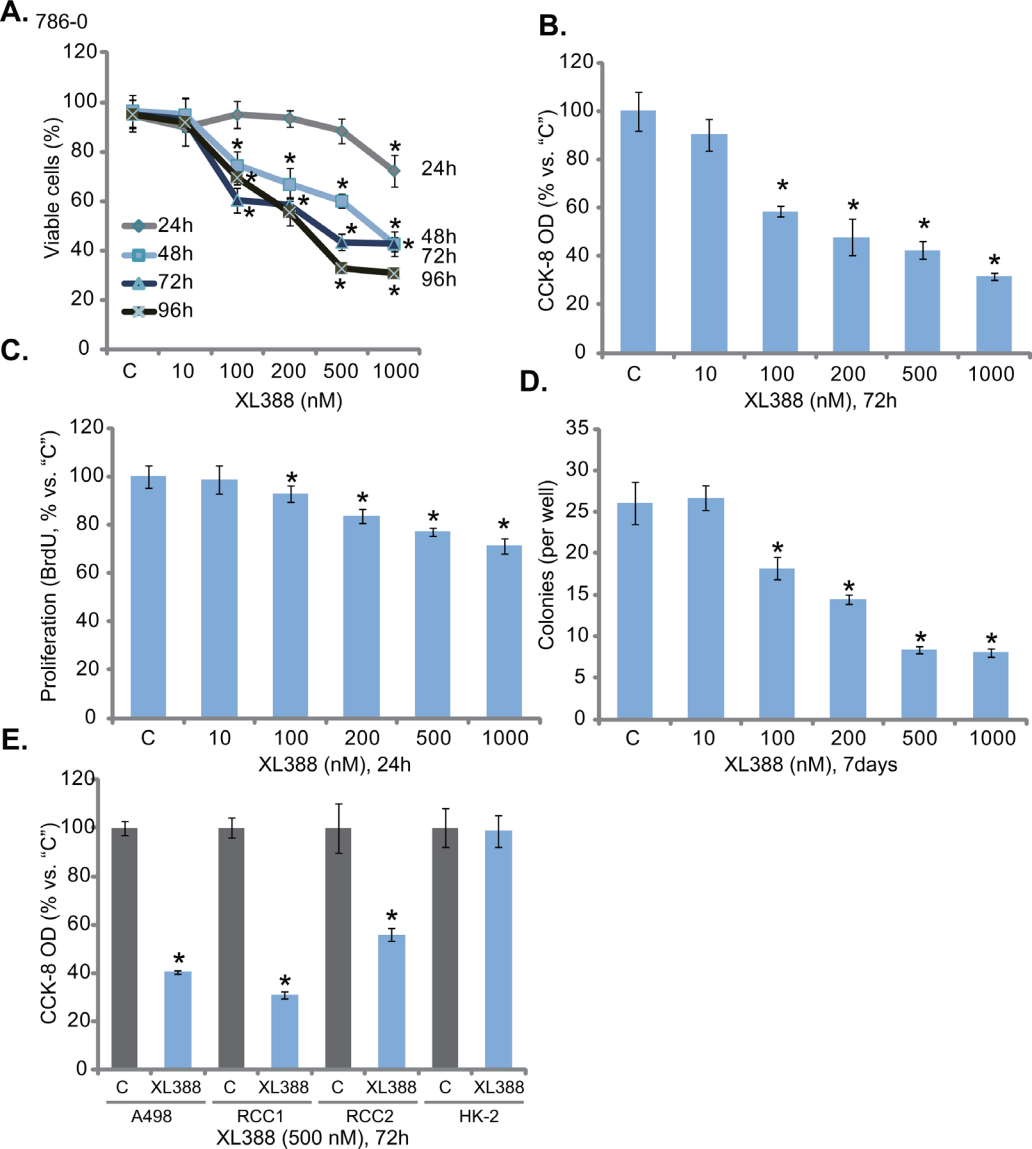
XL388 inhibits RCC cell survival and proliferation RCC cell lines (786-0 cells and A498 cells), the primary human RCC cells (two lines, “RCC1 and RCC2”) or the HK-2 proximal tubule epithelial cells were either left untreated (“C”, same for all figures) or stimulated with listed concentration of XL388, cells were further cultured in the conditional medium for applied time, cell survival **A**., **B** and **E**. and proliferation **C** and **D**. were tested by the assays mentioned in the text. For each assay, n=5. Data were always expressed as mean ± standard deviation (SD) (Same for all figures). Experiments in this figure were repeated four times, and similar results were obtained. *p < 0.05 vs. “C” group.

First, we evaluated the *in vitro* activity of XL388 in RCC cells. As demonstrated, 786-0 RCC cells, cultured in 10% FBS medium, were treated with XL388 at applied concentration. Trypan blue staining assay results demonstrated that XL388 dose-dependently induced 786-0 cell death (Figure 1A). Further, XL388 also displayed a time-dependent response in killing 786-0 cells (Figure 1A). Significant cell death was notified 48 hours after XL388 (100-1000 nM) treatment (Figure 1A). The IC50s of XL388 were 714.32 ± 66.19 nM, 351.26 ± 28.54 nM and 271.35 ± 15.37 nM after 48, 72 and 96 hours treatment (Figure 1A). Cell Counting Kit-8 (CCK-8) cell viability assay results in Figure 1B further demonstrated that XL388 was cytotoxic when added to the cultured 786-0 cells. XL388 again displayed a dose-dependent response in inhibiting 786-0 cells (Figure 1B).

The potential effect of XL388 on 786-0 cell proliferation was tested next. BrdU incorporation assay results in Figure 1C showed that XL388, at 100-1000 nM, significantly decreased BrdU ELISA OD, indicating the anti-proliferative activity by the compound. Similarly, 100-1000 nM of XL388 also dramatically decreased the number of proliferative 786-0 colonies (Figure 1D). Thus, XL388 was indeed anti-proliferation against 786-0 cells. Next, we studied XL388's activity in other RCC cells. As demonstrated, treatment with XL388 (500 nM, 72 hours) largely decreased the viability of A498 RCC cells [3, 4] and two primary human RCC cells (RCC1 and RCC2, Figure 1E). Intriguingly, same XL388 treatment was non-cytotoxic to the HK-2 proximal tubule epithelial cells [4, 25]. These results show that XL388 inhibits survival and proliferation of human RCC cells.

### XL388 activates apoptosis in RCC cells

**Figure 2:**
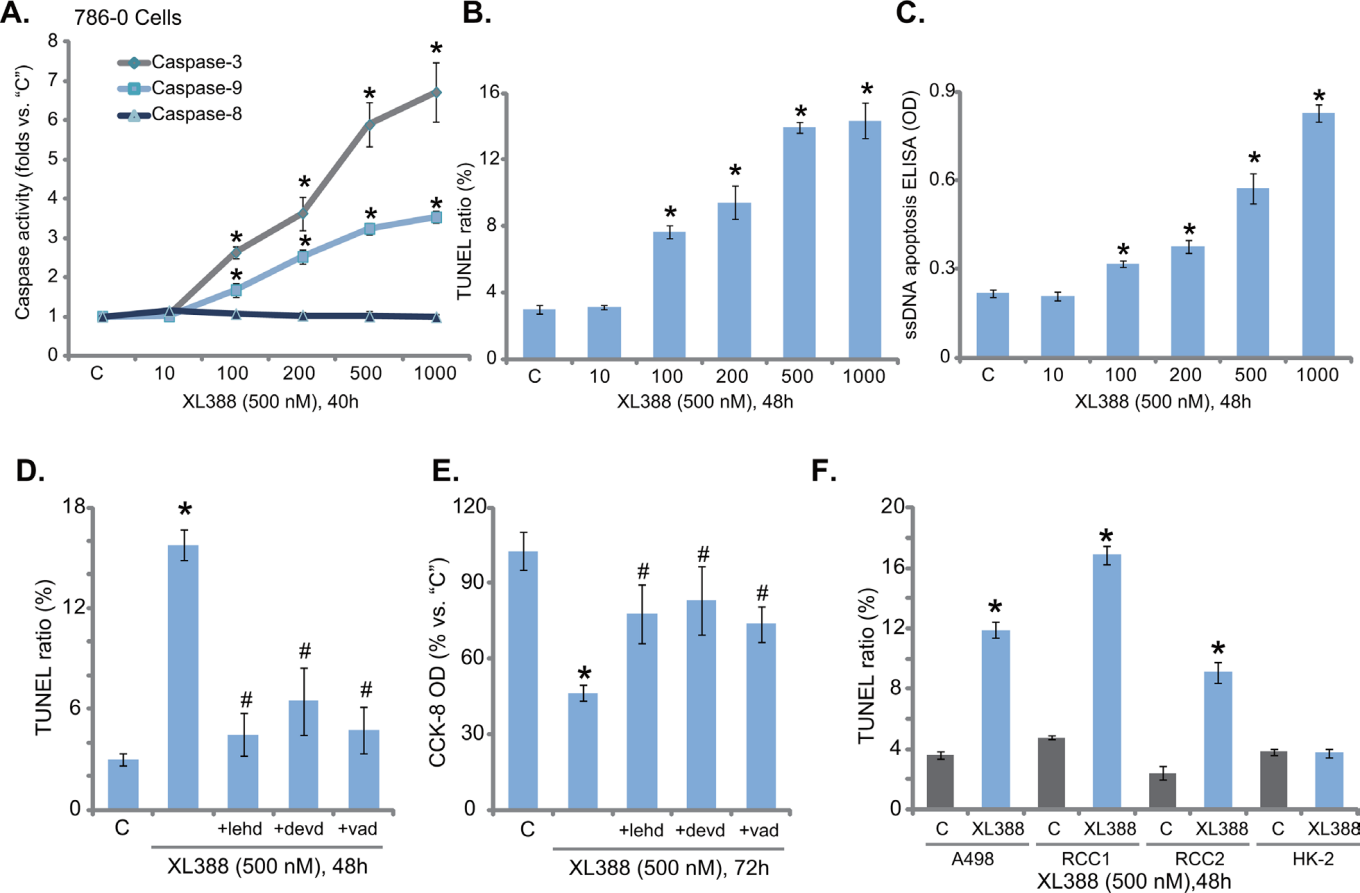
XL388 activates apoptosis in RCC cells 786-0 or A498 RCC cells, the primary human RCC cells (“RCC1 and RCC2”) or the HK-2 cells were stimulated with applied concentration of XL388, cells were further cultured in the conditional medium for applied time, cell apoptosis was tested by the caspase activity assay **A**., TUNEL staining assay **B** and **F**. and the ssDNA ELISA assay **C**. 786-0 cells were pre-treated for 30 min with 50 μM of the caspase-9 inhibitor z-LEHD-CHO (“+lehd”), the caspase-3 inhibitor z-DEVD-CHO (“+devd”) or the pan caspase inhibitor z-VAD-CHO (“+vad”), followed by XL388 (500 nM) treatment, cell apoptosis and viability were tested by the TUNEL assay **D**. and the CCK-8 assay **E**., respectively. For each assay, n=5. Experiments in this figure were repeated three times, and similar results were obtained. *p < 0.05 vs. “C” group. ^#^***p*** < 0.05 vs. “XL388” only group (D and E).

Next, the potential effect of XL388 on RCC cell apoptosis was tested. As shown in Figure 2A, treatment of XL388 in 786-0 cells dose-dependently increased the activity of caspase-3 and caspase-9, but not caspase-8. The latter is an indicator of extrinsic apoptotic pathway activation [26]. Meanwhile, the number of cells with TUNEL-positive nuclei was significantly increased following XL388 (100-1000 nM) treatment (Figure 2B), which also increased single-stranded DNA (ssDNA) apoptosis ELISA OD value (Figure 2C). These results clearly indicated that XL388 provoked apoptosis in 786-0 cells. To investigate the function of apoptosis in XL388-induced cytotoxicity, several caspase inhibitors were applied. Results showed that the caspase-9 inhibitor z-LEHD-CHO, the caspase-3 inhibitor z-DEVD-CHO and the pan caspase inhibitor z-VAD-CHO all largely inhibited XL388 (500 nM)-induced apoptosis activation (TUNEL assay, Figure 2D) and subsequent 786-0 cell lethality (Figure 2E, tested by the CCK-8 viability reduction). To test XL388's effect on apoptosis in other RCC cells, TUNEL staining assay was applied. Results showed that XL388 (500 nM) provoked significant apoptosis in A498 RCC cells and the two lines of primary RCC cells (Figure 2F). Yet, there was no significant apoptosis activation in XL388-treated HK-2 epithelial cells (Figure 2F). Collectively, these results show that XL388 provokes apoptosis in RCC cells.

### XL388 blocks mTORC1 and mTORC2 in RCC cells

**Figure 3:**
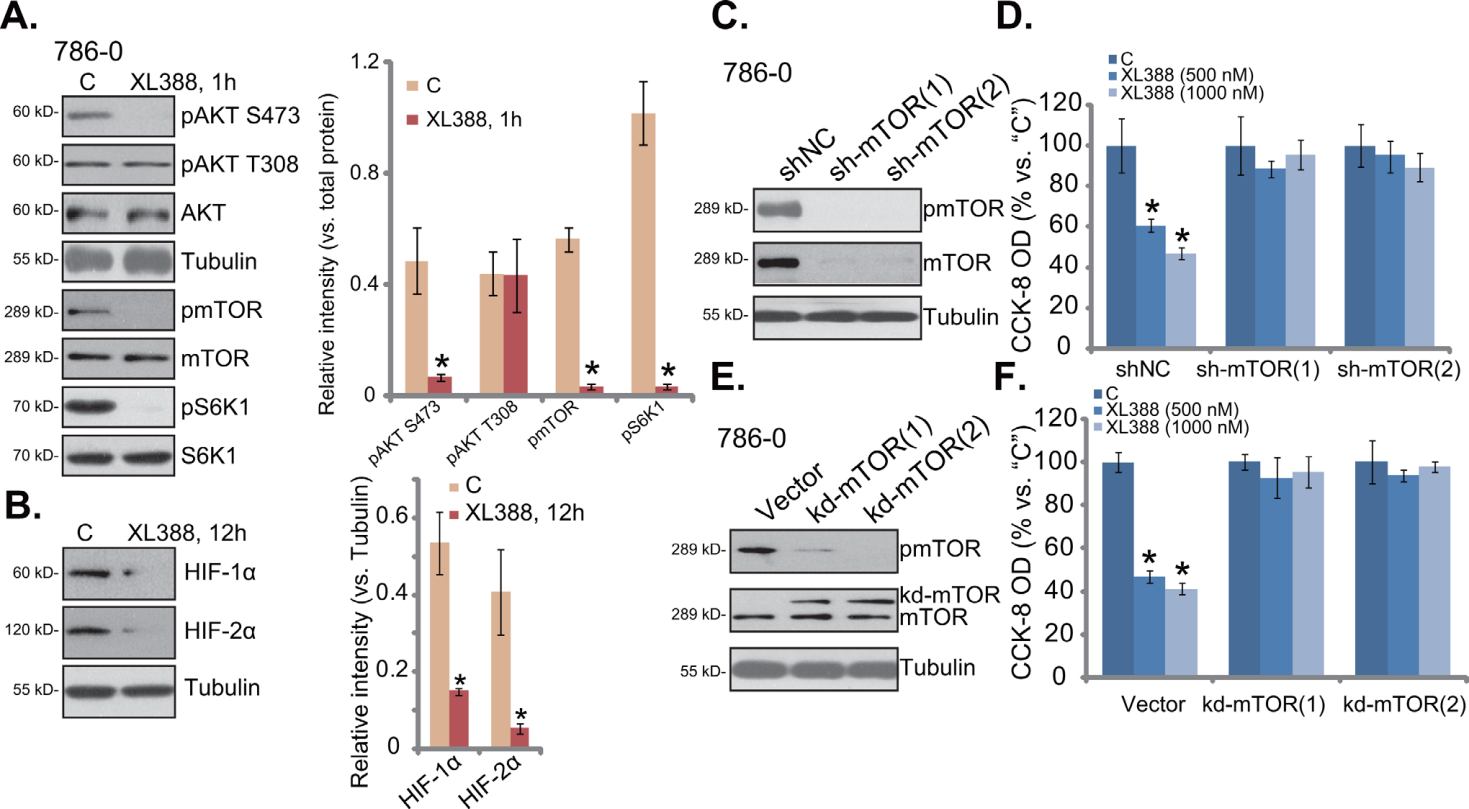
XL388 blocks mTORC1 and mTORC2 in RCC cells 786-0 cells were treated with XL388 (500 nM), cells were further cultured in the conditional medium for indicated time, expressions of listed proteins were shown **A-B**., blot results of three repeats were quantified); Expression of mTOR and tubulin in puromycin-selected 786-0 cells, expressing mTOR shRNA (“1/2”) or nonsense control lentiviral shRNA (“shNC”), as well as kinase-dead mTOR (“kd-mTOR”, Asp-2338-Ala, two lines) or empty vector (“Vector”, pSuper-puro), were shown **C** and **E**. Above cells were also treated with XL388 (500/1000 nM) for indicated time, relative cell survival (vs. “C”) was tested by CCK-8 assay **D** and **F**. For each assay, n=5. Experiments in this figure were repeated three times, and similar results were obtained. *p < 0.05 vs. “C” group.

We next tested mTOR signaling in XL388-treated RCC cells. Treatment with XL388 (500 nM) in 786-0 RCC cells led to almost complete inhibition of phosphorylated- (“p-“) mTOR (Ser-2448), p-S6K1 (Thr-389) and p-AKT (Ser-473) (Figure 3A), indicating concurrent inhibition of mTORC1 and mTORC2 [5, 6]. On the other hand, p-AKT (Thr-308) was not decreased by XL388 (Figure 3A). Notably, expressions of hypoxia-inducible factor 1α (HIF1α), a mTORC1-regulated gene [27], as well as HIF-2α, a mTORC2-regulated gene [3, 27], were both downregulated by XL388 in 786-0 cells (Figure 3B). Notably, expression of the above regular kinases was not changed by XL388 treatment in 786-0 cells (Figure 3A and 3B). These results suggest that XL388 blocks mTORC1/2 and downregulates HIF-1α/2α in 786-0 cells.

To confirm that mTOR inhibition is the primary reason RCC cell death by XL388, genetic methods were applied. First, two different mTOR shRNAs [“sh-mTOR (1/2)”] were applied. Both of them dramatically downregulated mTOR in 786-0 cells (Figure 3C). Intriguingly, in the mTOR-silenced cells, XL388 (500 and 1000 nM) was no longer cytotoxic (Figure 3D). Next, a kinase-dead mutation of mTOR (“kd-mTOR”, Asp-2338-Ala) [28] was introduced to 786-0 cells. Via puromycin selection, two stable 786-0 lines expressing kd-mTOR were established (Figure 3E). mTOR activation, tested by p-mTOR at Ser-2448, was blocked in kd-mTOR-expressing 786-0 cells (Figure 3E). More importantly, with mTOR-mutation, treatment with XL388 (500 and 1000 nM) was unable to kill 786-0 RCC cells (Figure 3F). These results together indicate that mTOR should be the primary target of XL388 in RCC cells.

### XL388 is more potent than rapalogs in killing RCC cells

**Figure 4:**
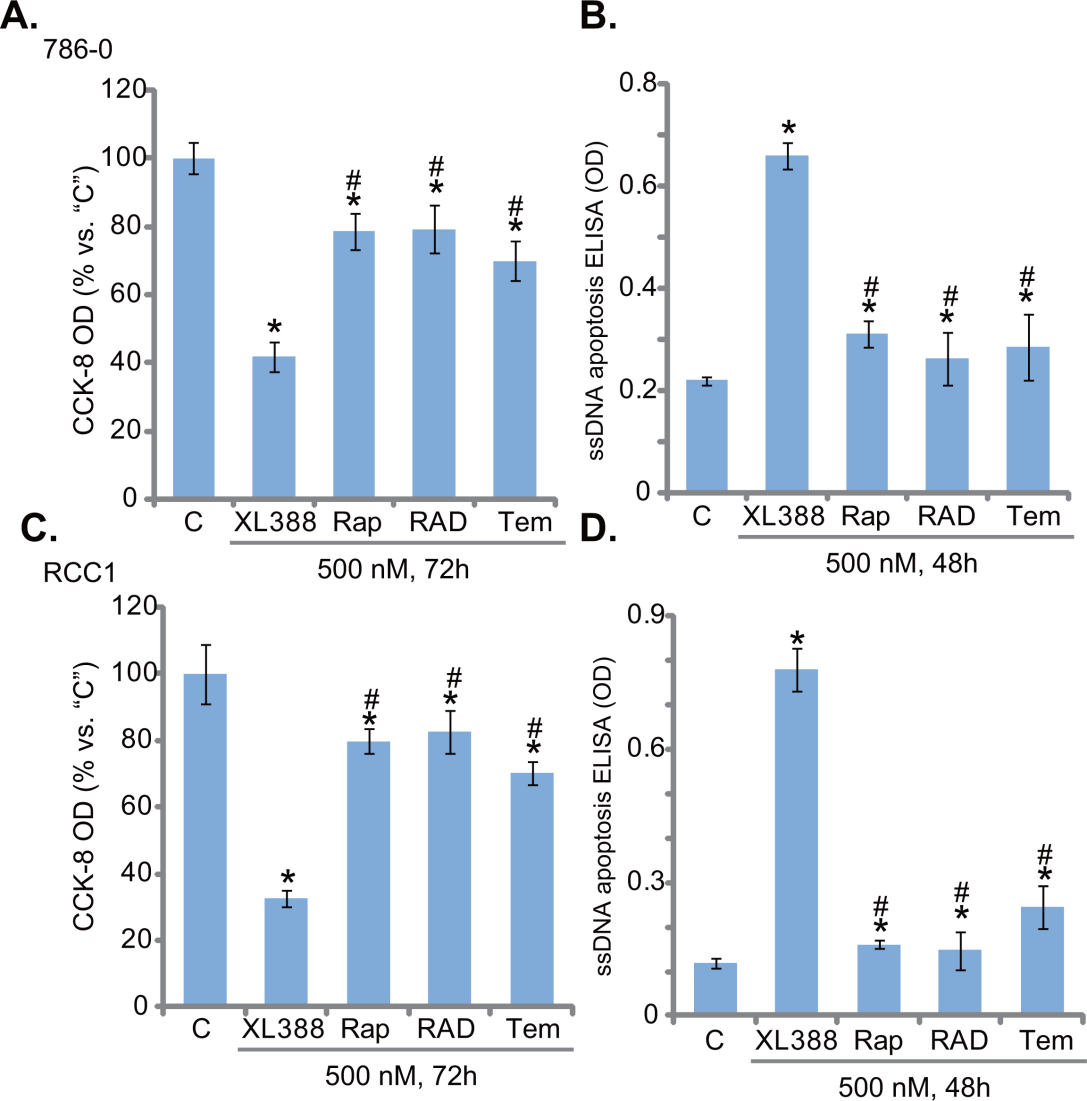
XL388 is more potent than rapalogs in killing RCC cells 786-0 cells or the primary human RCC cells (“RCC1) were treated with 500 nM of XL388, rapamycin (“Rap”), RAD001 (“RAD”, everolimus) or temsirolimus (“Tem”), cells were further cultured in the conditional medium for indicated time, cell viability and apoptosis were tested by the CCK-8 assay **A** and **C**. and ssDNA ELISA assay **B** and **D**., respectively. For each assay, n=5. Experiments in this figure were repeated three times, and similar results were obtained. *p < 0.05 vs. “C” group. ^#^***p*** < 0.05 vs. “XL388” only group.

The results above showed that XL388 blocked mTORC1 and mTORC2 activation simantanuously. We thus compared its cytotoxicity in RCC cells with known mTORC1 blockers or rapalogs [14]. As shown in Figure 4A and B, XL388 was significantly more potent in killing 786-0 cells than the same concentration (500 nM) of several rapalogs, including rapamycin, everolimus (RAD001) and temsirolimus. Similar results were also obtained in the primary RCC cells, where XL388 induced stronger survival reduction (Figure 4C) and apoptosis activation (Figure 4D) than the rapalogs. Thus, concurrent inhibition of mTORC1/2 by XL388 appears more potent than mTORC1 inhibition in killing RCC cells.

### MEK-ERK inhibition potentiates XL388-induced cytotoxicity against RCC cells

**Figure 5:**
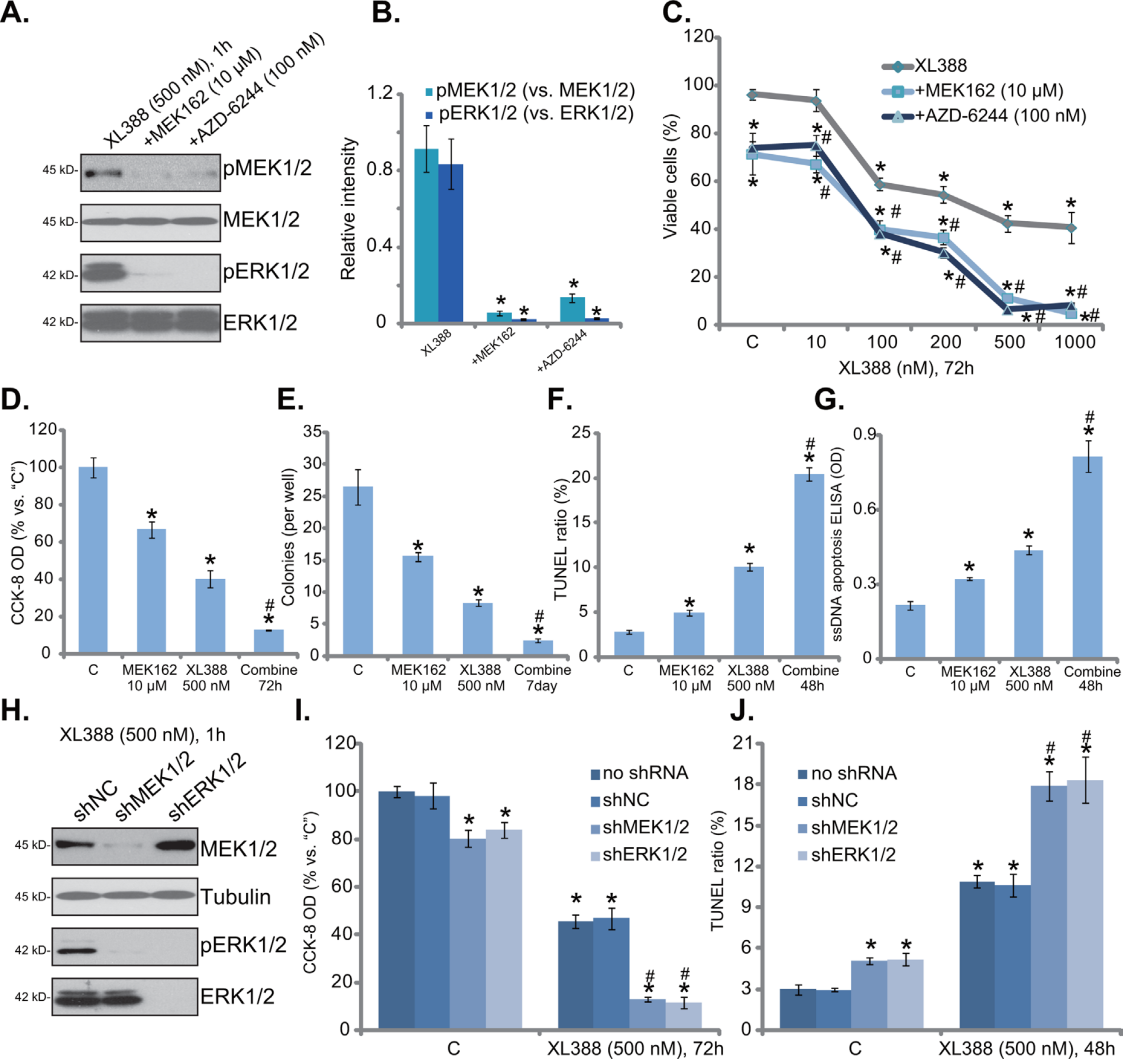
MEK-ERK inhibition potentiates XL388-induced cytotoxicity against RCC cells 786-0 cells were treated with applied concentration of XL388, or plus indicated MEK-ERK inhibitor, cells were further cultured in the conditional medium for designated time, expression of the labeled proteins was tested by Western blot assay **A**., blot results of three repeats were quantified in **B**. Cell death **C** and **D**. and proliferation **E**. were tested by the listed assays; Cell apoptosis was examined by the TUNEL staining assay **F**. and ssDNA apoptosis ELISA assay **G**. Stable 786-0 cells, expressing MEK1/2 shRNA (“shMEK1/2”), ERK1/2 shRNA (“shERK1/2”) or nonsense control shRNA (“shNC”), were treated with XL388 (500 nM), expression of listed proteins was shown **H**. Cell viability **I**. and apoptosis **J**. were also tested. For each assay, n=5. Experiments in this figure were repeated three times, and similar results were obtained. *p< 0.05 vs. “C” group. ^#^***p*** < 0.05 vs. “XL388” only group (C-G). ^#^***p*** < 0.05 vs. “shNC” group (I and J).

Existing evidences have suggested that several mTOR inhibitors could provoke feedback activation of oncogenic MEK-ERK signaling, which serves as a major resistance factor [29]. Others suggested that combined inhibition of mTOR and MEK-ERK cascades could achieve better anti-cancer activity than mTOR inhibition alone [29–32]. We therefore tested MEK-ERK signaling in XL388-treated cells. MEK-ERK inhibitors were applied, including MEK162 [33–35] and AZD-6244 [31, 36]. Both of them blocked MEK-ERK activation in 786-0 cells (Figure 5A, results were quantified in Figure 5B). More importantly, MEK162 and AZD-6244 significantly potentiated XL388-induced cytotoxicity in 786-0 cells (Figure 5C). The IC-50 of XL388, the concentration that killed 50% of 786-0 cells, decreased from over 300 nM to less than 30 nM with co-treatment of the MEK-ERK inhibitors (Figure 5C). Further studies showed that MEK162 also facilitated XL388-induced viability reduction (Figure 5D), proliferation inhibition (Figure 5E), and apoptosis (Figure 5F and 5G). Treatment of the MEK-ERK inhibitors alone also induced moderate 786-0 cell death and apoptosis (Figure 5C-5G). The combined activity was significantly potent than either single treatment (Figure 5C-5G).

The above pharmacological evidences suggest that MEK-ERK inhibition could potentiate XL388's cytotoxicity in RCC cells. Next, shRNA strategy was applied to knockdown MEK1/2 and ERK1/2 in RCC cells. As shown in Figure 5H, shRNA-mediated stable knockdown of MEK1/2 or ERK1/2 almost blocked ERK activation (p-ERK1/2) in 786-0 cells. Significantly, MEK1/2 or ERK1/2 silence also dramatically enhanced XL388-induced cytotoxicity, leading to a profound cell viability reduction (Figure 5I) and apoptosis activation (Figure 5J). Notably, MEK1/2 or ERK1/2 knockdown alone also induced minor but significant viability reduction (Figure 5I) and apoptosis (Figure 5J). Together, these results suggest that MEK-ERK inhibition potentiates XL388-induced cytotoxicity against RCC cells.

### XL388 inhibits 786-0 tumor growth *in vivo*, sensitized with co-administration of MEK162

**Figure 6:**
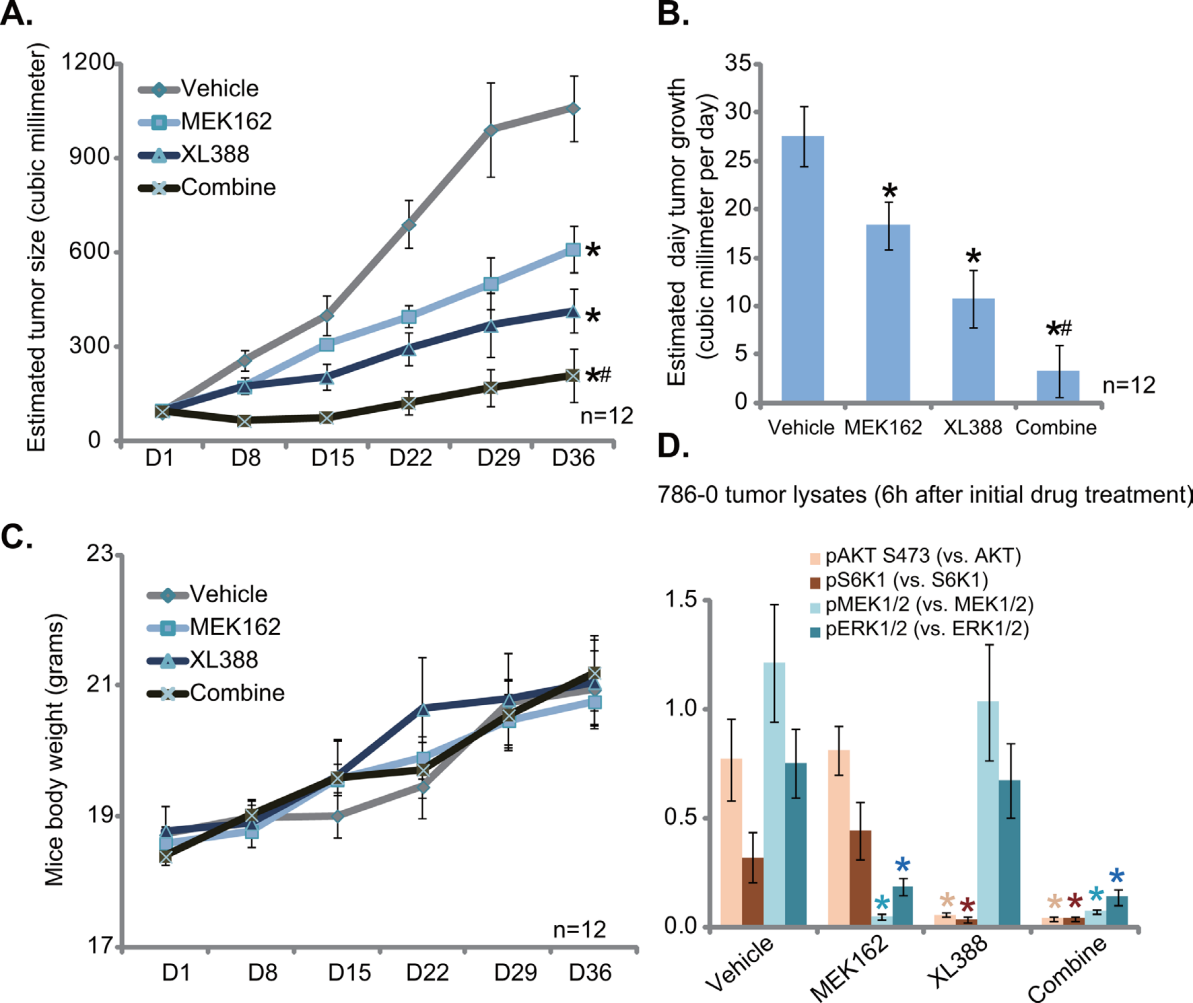
XL388 inhibits 786-0 tumor growth *in vivo*, sensitized with co-administration of MEK162 786-0 tumor-bearing nude mice (n=12 per group) were administrated with vehicle (saline), XL388 (20 mg/kg, oral gavage, every three days, 7 times), and/or MEK162 (2.5 mg/kg, lavage, once daily), tumor volumes (in mm^3^) was recorded weekly for 5 weeks **A**. Mice body weight (in gram) was also recorded **C**. Estimated daily tumor growth (in mm^3^ per day) was calculated **B**. Six hours after initial drug treatment, xenografted tumors were isolated (one mice per group), and tissue lysates were subjected to Western blot assay of listed proteins **D**., blot results of three repeats were quantified). *p < 0.05 vs. “Vehicle” group. ^#^ *p* < 0.05 vs. XL388 only group.

At last, we tested the potential anti-RCC activity of XL388 *in vivo*, using a 786-0 cell xenograft tumor model [3, 4]. 786-0 cells were injected to the flanks of the nude mice. Within three weeks, the xenograft tumors were established with the initial tumor size around 100 mm^3^ [3, 4]. Tumor growth curve results in Figure 6A demonstrated that oral administration of XL388 (20 mg/kg, every three days, × 7 times) [24] significantly inhibited 786-0 tumor growth in nude mice. Remarkably, co-administration with MEK162 (2.5 mg/kg, lavage, once daily) [37], the MEK-ERK inhibitor, dramatically potentiated XL388's anti-tumor activity (Figure 6A). The XL388 plus MEK162 co-administration led to profound 786-0 tumor inhibition, more potently than either single treatment (Figure 6A). Daily tumor growth results in Figure 6B further confirmed that MEK162 facilitated XL388-induced anti-tumor activity *in vivo* (Figure 6B). Interestingly, the mice body weight, which is the indicator of general mice health, was not significantly changed between each group (Figure 6C), suggesting that these mice were well-tolerated to the regimens tested here. When analyzing tumor tissue samples, we showed that XL388 plus MEK162 co-administration led to concurrent inhibition of MEK-ERK and mTORC1 (indicated by p-S6K1)/mTORC2 (indicated by p-AKT Ser-473) (Figure 6D, blot results of three repeats were quantified). On the other hand, each single treatment only achieved inhibition of one pathway (Figure 6D). Together, we show that MEK162 sensitizes XL388-induced anti-RCC activity *in vivo*.

## DISCUSSION

mTOR hyper-activation is often observed in RCC [10, 38]. It has been shown that the two mTOR complexes, mTORC1 and mTORC2, are important for many cancerous behaviors of RCC [6, 14]. The anti-cancer activity by the first generation of mTOR inhibitors, or rapalogs, is generally limited, as they only block mTORC1, but not mTORC2 [39]. Further, rapalogs-induced mTORC1 inhibition often induces feedback activation of pro-cancerous signaling cascade, including PI3K-AKT and ERK-MAPK [29, 39]. Due to these reasons, the mTOR kinase inhibitors, also known as the second generation of mTOR inhibitors, were developed [15].

In this study, we showed that XL388, a potent mTOR kinase inhibitor [23, 24], inhibited survival and proliferation of both established and primary human RCCs, possibly via inducing caspase-dependent apoptosis. At the molecular level, XL388 concurrently blocked activation of mTORC1 and mTORC2. We propose that mTOR is the primary target of XL388. And XL388 was non-effective in RCC cells with mTOR knockdown or kinase-dead mutation. Importantly, XL388 was significantly more potent than rapalogs (rapamycin, everolimus and temsirolimus) in killing RCC cells. *In vivo*, XL388 oral administration inhibited 786-0 RCC tumor growth in nude mice. These preclinical results indicate the translational value of this mTOR kinase inhibitor for RCC treatment.

Studies have demonstrated a strong correlation between inactivation of von Hippel-Lindau protein (pVHL) and RCC's poor prognosis [40]. Epidemiological studies revealed that over 50% of sporadic RCC patients have somatic VHL mutations [41]. pVHL is the E3 ubiquitin ligase for HIF-1α/2α [41]. Its inactivation or mutation would cause HIF-1α/2α accumulation and activation, leading to vascular endothelial growth factor (VEGF) expression and tumor angiogenesis [41]. Recent studies have proposed that HIF-2α is even more important than HIF-1α in RCC tumorigenesis [42]. One important finding of this study is that treatment of XL388 in RCC cells led to downregulation of both HIF-1α and HIF-2α. This should have clinical significance for RCC.

Another key finding of this study is that MEK-ERK could be a key resistance factor of XL388 in RCC cells. MEK-ERK inhibitors (MEK162 and AZD-6244) or shRNA knockdown of MEK1/2-ERK1/2 dramatically sensitized XL388-induced killing of RCC cells. *In vivo*, XL388-mediated anti-tumor activity was further sensitized with co-administration of MEK162. It would be interesting to test the underlying signaling mechanisms of MEK-ERK inhibition-mediated XL388 sensitization. Further studies will also be needed to explore the same phenomenon in other cancer cells.

## MATERIALS AND METHODS

### Chemicals and antibodies

XL388 was provided by MedChem Express (Shanghai, China). Rapamycin, everolimus (RAD001), temsirolimus, MEK162 and AZD-6244 were obtained from Selleck (Shanghai, China). The caspase-9 inhibitor z-LEHD-CHO, the caspase-3 inhibitor z-DEVD-CHO and the pan caspase inhibitor z-VAD-CHO were obtained from Invitrogen (Shanghai, China). Antibodies of p-AKT (Ser 473, #9271), p-AKT (Thr 308, #9275), AKT (9272), p-p44/42 MAPK (p-ERK1/2, #9101), ERK1/2 (#9102), p-MEK1/2 (#9121), MEK1/2 (#9122), p70-S6 Kinase (S6K1 #9202), p-S6K1 (Thr389, #9205), mTOR (#2983), p-mTOR (#2971), HIF-1α (#3716), HIF-2α (#7096), and (β-) tubulin (#2128) were all obtained from Cell Signaling Tech (Shanghai, China).

### Cell culture

The established human RCC cell lines (786-0 and A498) and HK-2 human proximal tubule epithelial cell line were provided by Dr. Zheng's Group at Nantong University [3, 4, 43]. Cells were cultured as described [3, 4, 43]. Trypan blue staining was applied, and only dead cells with compromised plasma membrane would take trypan blue. Thus, trypan blue negative cells were recorded as viable cells.

### Primary RCC cells

Fresh RCC specimens were obtained from two different RCC patients (RCC1, male, 55 years old; and RCC2, female, 45 years old) with nephroureterectomy. The two patients were enrolled at Huashan Hospital Affiliated to Fudan University (Shanghai, China), both received no treatment prior to surgery. RCC tissues were thoroughly washed and then minced into small pieces, which were then digested via collagenase I for 30 min. Afterwards, primary cancer cells were pelleted and washed, and then cultured in the described DMEM medium [3]. Primary RCC cells of passage 3-6 were utilized for experiments. Protocols requiring human tissues were approved by the Ethics Review Board (ERB) of authors institution. The written-informed consent was obtained from each RCC patient. All investigations were conducted with the principles expressed in the Declaration of Helsinki.

### CCK-8 assay of cell viability

Following treatment, cell viability was evaluated via the Cell Counting Kit-8 (CCK-8, Dojindo Laboratories, Kumamoto, Japan) assay with the manufacturer’s instructions. Absorbance was measured at 490 nm through a Microplate Reader.

### Clonogenic assay

786-0 cells with indicated treatment were plated onto 6-well plate at 2000 cells per well. Following incubation of 7 days, the remaining large proliferative colonies were fixed, stained, and counted manually.

### BrdU incorporation assay

To assay of cell proliferation, BrdU ELISA assay kit (Cell Signaling, Nanjing, China) was used [44]. The ELISA OD value of treatment group was always normalized to percentage of untreated control group.

### Apoptosis quantification by the single-stranded DNA ELISA assay

After indicated treatment, cells were subjected tothe single-stranded DNA (ssDNA) apoptosis ELISA (Chemicon International, Temecula, CA) assay. The detailed protocol can be seen in other studies [45, 46].

### Assay of caspase activity

After treatment, 20 μg protein lysates (per treatment) were added to caspase assay buffer [3] along with the corresponding caspase substrate (Calbiochem, Darmstadt, Germany). The AFC (7-amido-4-(trifluoromethyl)-coumarin) release was then quantified via a Fluoroskan system [3].

### TUNEL staining assay

Cell apoptosis was examined by the TUNEL staining assay [47]. Percentage of TUNEL positive nuclei was calculated from at least 200 cells per treatment in five independent experiments.

### Western blot assay

Cells or tumor tissues were lysed by the lysis buffer described [3]. The quantified lysates (30 μg per lane) were separated by SDS-PAGE gels, and were transferred to the PVDF membrane. After incubation in the specific primary antibody and corresponding secondary antibody, the targeted protein band was visualized via an enhanced chemiluminescence (ECL) detection kit (Amersham, Shanghai, China).

### shRNA and stable cell selection

MEK1/2 shRNA, MEK1/2 shRNA and two mTOR lentiviral shRNAs were purchased from Santa Cruz Biotech (Shanghai, China), which contain a puromycin resistance gene [48]. RCC cells were cultured in the presence of polybrene (2.0 μg/mL), the lentiviral-shRNA (10 μL/mL medium) were directly added to the cells for 24 hours. Cells were then culture in fresh complete medium for another 12 hours. Stable RCC cells were selected by puromycin (5 μg/mL, Sigma) for 4 days. Control cells were incubated with nonsense control lentiviral shRNA (“shNC”, Santa Cruz). Expression of shRNA-targeted protein in the stable cells was tested by Western blot assay.

### mTOR kinase-dead mutation

The kinase-dead mTOR (“kd-mTOR-flag”, Asp-2338-Ala) construct and the empty vector (pSuper-puro) were from Dr. Liu's group [28]. The construct was transfected into RCC cells by Lipofectamine 2000 (Invitrogen). Cells were then subjected to puromycin (5.0 μg/mL, Sigma) selection for 4 days. Expression of the kd-mTOR in stable cells was confirmed by Western blot assay.

### Tumor growth in nude mice

As reported [4], female nude mice (7-8-week old, 18-20 g in weight) were purchased from Animal Center of authors institution. For each mouse, 5 × 10^6^ 786-0 cells were injected into the left flank. Within three weeks, the xenograft tumors were established. Mice were divided into four groups. Mice body weight and bi-dimensional tumor measurements were recorded every 7 days for a total of 35 days. Tumor volume was estimated using the standard formula: (length × width^2^)/2. All animal protocols were approved by the authors' institution IACUC.

### Statistical analyses

Data were always expressed as mean ± standard deviation (SD). Statistical analyses were performed via one-way analysis of variance (ANOVA, SPSS 16.0). IC-50 was calculated by SPSS 16.0 using a sigmoidal dose-response curve model. Multiple comparisons were performed using Tukey’s honestly significant difference procedure.
